# Supplementary figures and images for: Development of a size-separation technique to isolate Caenorhabditis elegans embryos using mesh filters
Source: PLoS One. 2025 Apr 24;20(4):e0318143. doi: 10.1371/journal.pone.0318143 (PMC12021249; doi:10.1371/journal.pone.0318143)

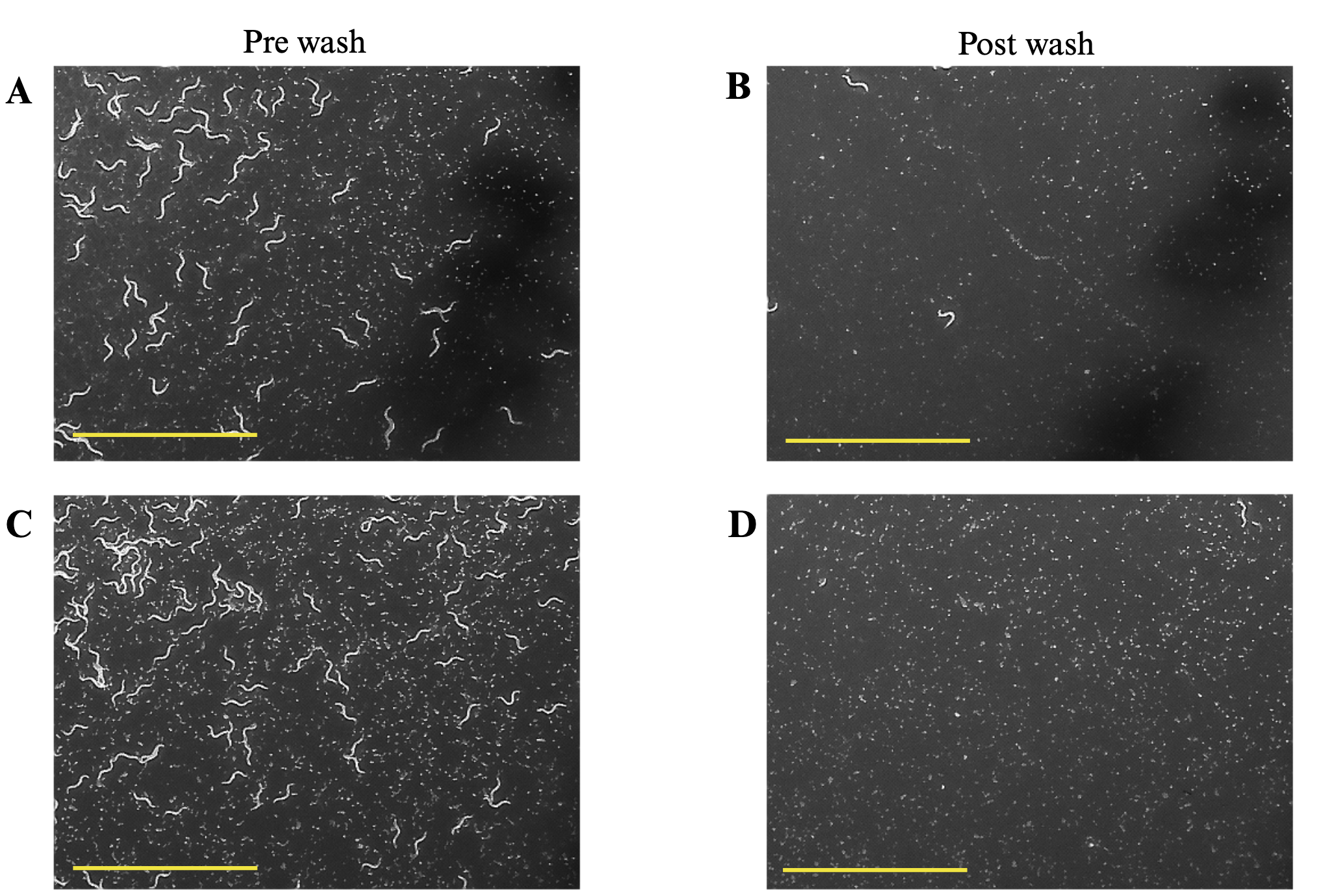

Supplement: S1 Fig — (A) A 6 cm HB101-seeded plate before washing with M9 buffer. (B) A 6 cm HB101-seeded plate after washing with M9 buffer. (C) A 6 cm OP50-seeded plate before washing with M9 buffer. (D) A 6 cm HB101-seeded plate after washing with M9 buffer. The yellow line is a 1 cm scale bar. The figure was modified using Biorender. (TIF) [file pone.0318143.s001.tif]

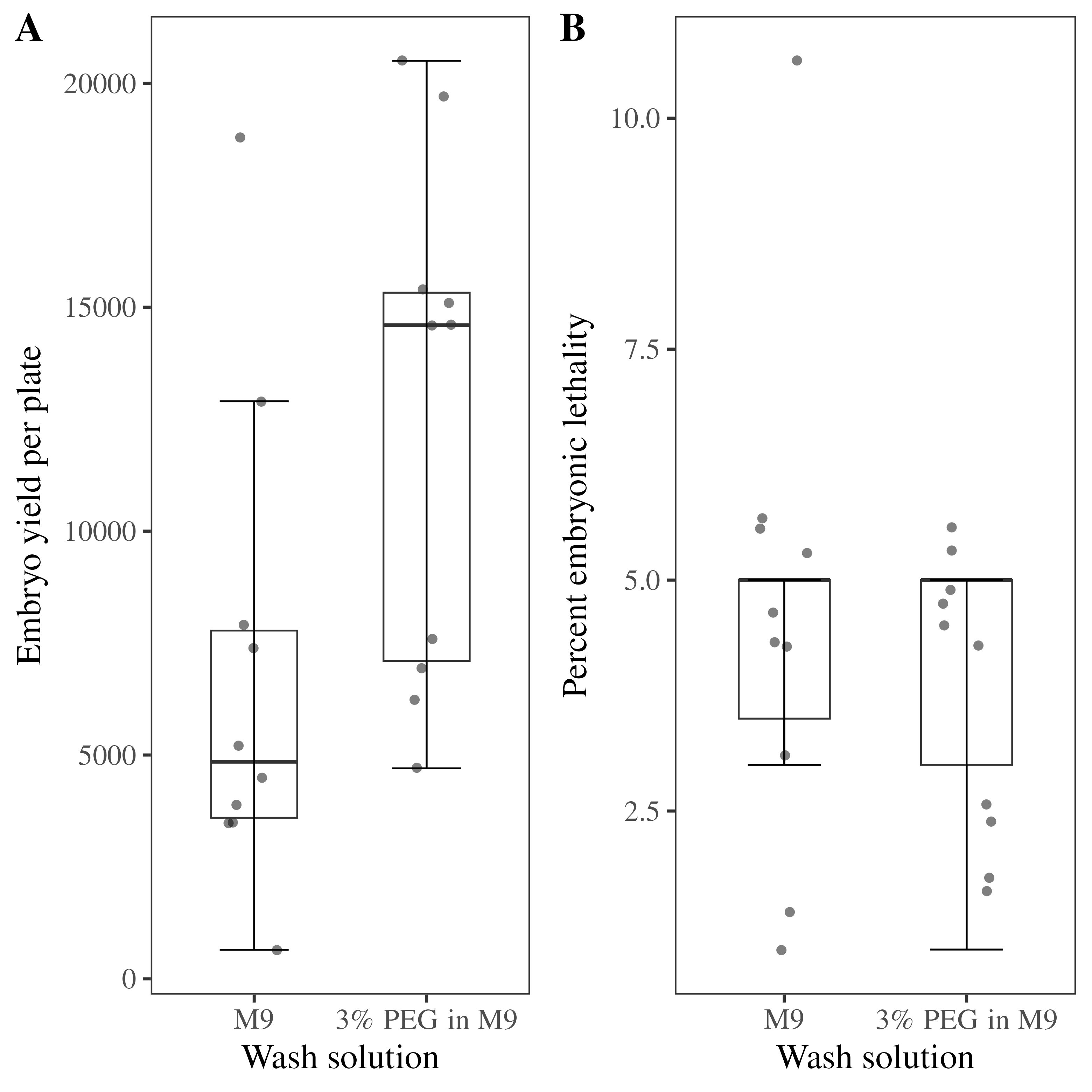

Supplement: S2 Fig — (A) Box plots of G1 embryo counts obtained from one 6 cm OP50-seeded plate using M9 buffer or 3% PEG in M9 buffer as the subsequent wash solutions. Median is represented by the solid horizontal line with the 75th and 25th quartiles on the top and bottom of the box, respectively. Whiskers extend from the box to the maximum and minimum values that fall within 1.5 times the interquartile range above and below the 75th and 25th percentiles, respectively. Significantly higher numbers of G1 embryos were collected when 3% PEG was added to the M9 buffer (p = 0.026, Wilcoxon rank-sum test). (B) Box plot of percent G1 embryonic lethality measured 24 hours after collecting the G1 embryos using M9 buffer or 3% PEG in M9 buffer as the subsequent wash solutions. The median is represented by the solid horizontal line with the 75th and 25th quartiles on the top and bottom of the box, respectively. Whiskers extend from the box to the maximum and minimum values that fall within 1.5 times the interquartile range above and below the 75th and 25th percentiles, respectively. No significant difference in percent embryonic lethality was observed (p = 0.58, Wilcoxon rank-sum test). A total of 10 replicates were tested by each of two experimentalists. (TIF) [file pone.0318143.s002.tif]

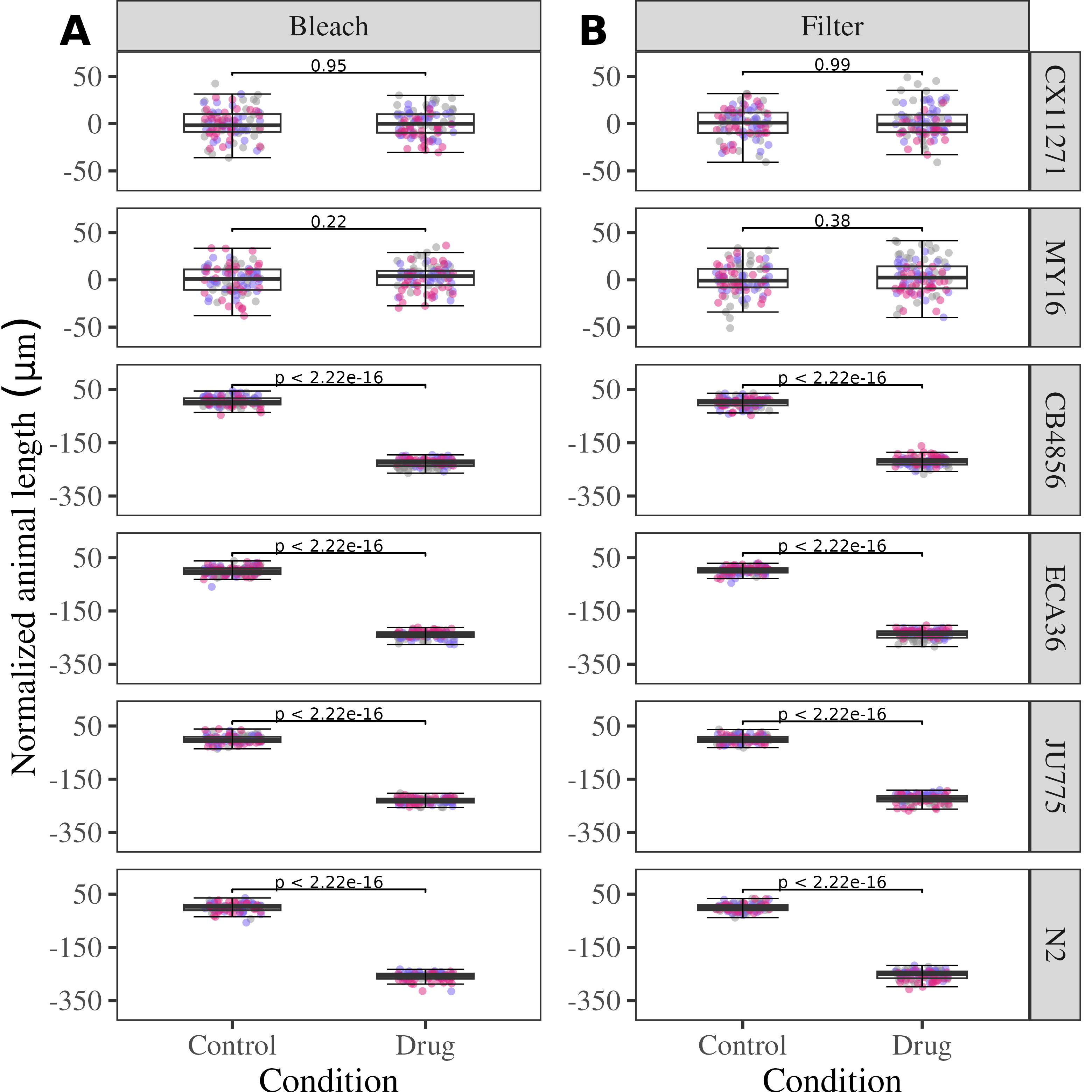

Supplement: S3 Fig — Box plots showing normalized animal length for larvae developed from G1 embryos obtained by bleach synchronization or filtration protocols for six C. elegans strains. Median animal lengths of strains exposed to 30 μM albendazole were normalized to the mean of all median animal lengths for the control DMSO condition. The median is represented by the solid horizontal line with the 75th and 25th quartiles on the top and bottom of each box, respectively. Whiskers extend from each box to the maximum and minimum values that fall within 1.5 times the interquartile range above and below the 75th and 25th percentiles, respectively. Each point is the summarized measurement of an individual well containing between five and 30 animals. The box plot is faceted by the method by which the G1 embryos were obtained (bleach or filtration). The sensitivity of the strains to the drug remained consistent between the bleach and filtration methods, as indicated by the p-values (Wilcoxon rank-sum test) in each facet. The assay was replicated three times, as indicated by the colors of the points. (TIF) [file pone.0318143.s003.tif]
