# Supplementary material for: Development of a size-separation technique to isolate Caenorhabditis elegans embryos using mesh filters
Source: PLoS One. 2025 Apr 24;20(4):e0318143. doi: 10.1371/journal.pone.0318143 (PMC12021249; doi:10.1371/journal.pone.0318143)
Supplement: S1 Table — Exposing the G1 embryos (and a few larvae) recovered from the 20 μM filter to 25% bleach solution for 30 seconds killed the larvae and had a minimal effect on the embryos as measured by determining the embryonic lethality after 24 hours. Data were collected from one replicate for conditions 1–5. Because living larvae were observed for the first four conditions and the percent embryonic lethality was greater than 50% for the fifth condition, additional replicates were not performed. Data were collected from five replicates for the sixth and seventh conditions. (PDF) [file pone.0318143.s004.pdf]

S1Table. Optimization of filtration conditions.
Exposing the G_1 embryos (and a few larvae) recovered from the 20 μM filter to 25% bleach solution for 30 seconds killed the larvae and had a minimal effect on the embryos as measured by determining the embryonic lethality after 24 hours. Data were collected from one replicate for conditions 1 to 5. Because alive larvae were observed for the first four conditions and the percent embryonic lethality was greater than 50% for the fifth condition, additional replicates were not performed. Data were collected from five replicates for the sixth and seventh conditions.

Condition number	Percent bleach solution	Incubation time in seconds	Alive larvae observed	Percent embryonic lethality measured after 24 hours
1	10	120	Yes	9
2	20	60	Yes	12
3	20	120	Yes	12
4	20	150	Yes	45
5	25	150	No	60
6	25	60	No	34.4
7	25	30	No	13.3
